# Miscellany

**Published:** 1901-09

**Authors:** 


### Miscellany.

To repair Broken Plaster Models.—To repair broken models, use Diamond Magic Invisible Cement, put up in small vials by the Diamond Ink Manufacturing Company, Milwaukee, Wis. It is a clear liquid, and models may be repaired so that the break will hardly be visible. Broken impressions may be put together in the same way. The cement thickens with age and exposure, and I know of no way to thin it, but it costs only ten cents a bottle, which is enough to repair two or three hundred models, so that it is a small item. Models repaired in this way will stand the pressure of closing the flask and vulcanizing very nicely.—F. H. Roberts, *Dental Brief.*

[The writer has repaired models with a thickly mixed oxyphosphate cement, which has answered every purpose.]

---

Pyorrhœa Alveolaris in Dogs.—My attention has been called to one or two dogs lately, highly domesticated dogs that live in the house and sleep on a rug, which are losing their teeth from pyorrhœa. They eat a good deal of cake and candy and such things. The canine family, as a rule, usually have pretty good teeth, but I speak particularly of the highly domesticated dog. In one of the dogs of which I speak the central incisors have been lost by the disease. These dogs, as I say, live very largely on sweetmeats.—Dr. J. G. Reid, *Western Dental Journal.*

---

The Use of Logan Crowns as Piers in Bridge-Work.—Dr. A. E. Peck, at the Annual Meeting of the Minnesota State Dental Society, gave a bench clinic in which he demonstrated the use of the Logan crown as a pier in bridge-work. The root and cap were prepared after the same manner as for a Richmond crown. He then punched a hole for the reception of the pier. A piece of pure gold was placed between the porcelain and the cap to obtain perfect adaptation. A piece of pure gold, 32-gauge, was then burnished on the back of the Logan crown, extending to the gold band which formed a backing for the same.—*Dental Review.*

Fetid Breath.—Professor B. Fränkel (*Archiv. für Laryngol.*) offers some remarks upon the subject of disagreeable odors arising from the mouth and other parts of the respiratory tract. In order to determine definitely whether the odor arises from the nose or the mouth, a piece of card-board of suitable dimensions is held against the upper lip beneath the nose, and the patient, with the mouth closed, blows first through one nostril and then through the other. The observer sits with his nose at the opposite end of the card-board, and may in this way determine whether one or both nostrils afford the source of the odor. Then, with the nostrils closed, the patient is allowed to breathe through the mouth. If one has determined that the odor arises from the mouth, it is necessary to further decide whether any particular portion of the mouth or pharynx is responsible for the odor. To accomplish this, Fränkel collects the secretion on a cotton applicator from each suspected spot, and subjects it to his olfactory judgment.

If carious teeth are responsible for the fetor, the dentist can remove the odor completely. He finds the tonsils frequently the seat of the odor, due to decomposition of their secretion or caseous abscesses in the tonsils. Where the secretions of the entire mucous membrane have undergone fetid decomposition, or where the disagreeable odor arises from the lower respiratory tract or the œsophagus, the prospect of treatment is not good. In any case, he recommends a bactericidal and deodorizing mouth-wash.—*Dental Digest.*

